# A Treatise on Tetanus, Illustrated by a Number of Cases

**Published:** 1817-07

**Authors:** 


*A Treatise on Tetanus, illustrated by a Number of Cases.* By JOHN MORRISON, M. D. One vol. 8vo. pp. 122.

Dr. MORRISON was led to compose the present Treatise on Tetanus, from having had considerable experience in that disease, during an eight years practice in the Colony of Demerara, where it is of frequent occurrence. The land of this part of the South American Continent is low, flat, and marshy, abounding with swamps, and, with the exception of a stripe along the banks of the Demerari, is covered with trees of various dimensions, whose roots, for a great part of the year, lie bedded in water. The prevalent diseases are intermittents, fever, hepatitis, enteritis, rheumatism, dysentery, and, among children, hydrocephalus.

Although tetanus occurs in all climates, yet it is infinitely more predominant where heat and moisture prevail. If negroes be more liable to its attacks than whites, it is probably owing, in our author's opinion, to their being so constantly exposed to the remote and exciting causes, rather than to any peculiar predisposition in their system. In Europe the traumatic species is almost the only form in which it occurs; whereas, between the tropics, the idiopathic tetanus is by no means unfrequent. No particular *texture* appears more liable to produce tetanic affections, when wounded, than another; and the same may be said of wounds themselves.

We cannot follow Dr. M. through his symptomatology of tetanus, which, though excellent and appropriate in a professed treatise, requires no analytical notice in this place, excepting where the author's observations are novel or interesting.

Dr. M. does not look upon tetanus, even the traumatic form, as so very dangerous a disease, in tropical climates, as authors have represented it. He has witnessed many instances of recovery both from traumatic and idiopathic tetanus, and, strange as it may appear, the instances of cure in the *former* have been nearly as numerous as in the *latter*. In upwards of twenty cases of this disease which he witnessed among negroes, the pulse was in no instance, accelerated in the manner related by Dr. Parry. He has never known it above 98, whether the termination was favourable or fatal. The following prognostic passage we shall transcribe.

"When the disease comes on gradually; when for the first three or four days the muscles of the jaws are solely affected, and that perhaps not in any alarming degree; when the abdomen is not p eternaturally hard, or the bowels obstinately costive; when the skin is moist and moderately warm, and above all, when the patient enjoys sleep, we may (by the means hereafter to be spoken of) entertain strong hopes of an eventual recovery. An increased flow of saliva where mercury has, or has not been used, is always to be regarded as favourable; the less the general air of the countenance is changed, the better. On the other hand, when the attack is violent and sudden; when the muscles of the neck, back and abdomen, are rigidly contracted; when the patient complains of a shooting pain from the sternum towards the spine; when the belly feels hard like a board, and the least pressure thereon produces spasmodic twitchings or contractions of the muscles of the neck, jaws, &c.; or when the same effect is brought about by the presentation of any substance (solid or fluid) near the mouth, we have much reason to fear a fatal termination. Spasmodic startings of the muscles set in sometimes early in the disease, and recurring every eight or ten minutes, are to be regarded as very unfavourable." p. 29.

The only disease which tetanus can be confounded with, is rabies contagiosa. In the latter, however, there is generally fever; frequently increased heat of the body. In rabies contagiosa, vomiting is common at the commencement; not so in tetanus. The delirium too of hydrophobia is absent in tetanus. The shooting pain from the sternum to the spine is seldom wanting in tetanus or present in the other.

Dr. M. asserts, that the bodies of tetanic patients run rapidly into putrefaction after death. Dissection has thrown little, if any light on the nature of the disease. In fact, in many most careful and rigid anatomical investigations *post mortem*, no morbid appearance whatever could be discovered either in the head, thorax, or abdomen, hence the organic derangement *sometimes* found may be fairly presumed to be accidental—not occasional. That tetanus is considerably under atmospherical influence may be inferred from this circumstance, that some plantations in Demerara, of two or three hundred slaves, know the disease only by name; whilst in others, it might be almost said to be endemial. The trismus infantum is invariably fatal when occurring on or before the ninth day.

*Treatment of Tetanus.* Dr. M. believes, that spontaneous cures do occasionally take place in tropical climates. One decided instance of traumatic tetanus giving way to the efforts of Nature fell under his own observation. The treatment of idiopathic and symptomatic tetanus is considered the same. For although it is common and proper in the West Indies to apply some stimulating substances as ol. terebinth. or the like, to recent wounds, together with emollient cataplasms, so as to induce free suppuration, yet when constitutional tetanic symptoms have once commenced, there is little or no dependence on local treatment. By way of prevention, Dr. Clarke advises a slight mercurial ptyliasm to be brought on after wounds in hot climates, or under suspicious circumstances. For the same purpose, the complete division of half divided nerves, tendons, &c. might be proper. The Spanish physicians bathe the wound, for an hour or more, in warm oil, while some subsequently apply lunar caustic, superacetate of lead, &c. The principal general remedies that have been recommended are, the cold affusion, mercury, opiates, wine and bark, the warm bath, cathartics, blisters, anti-spasmodics. We shall not stop to notice the history of each of these remedies, but give the substance of Dr. Morrison's own remarks and experience. During the Doctor's first three years residence in Demerara, and in the first eight or ten cases, the *cold affusion* was invariably used, but with so little success that it was ultimately left entirely off, and the warm bath substituted.

*Mercury.* Spontaneous salivation has often been observed in tetanic patients whose cases terminated favourably, hence probably the first idea of using mercury. In hot countries tetanus is seldom so rapid as to prevent the introdnction of mercury in quantity sufficient to salivate, before the disease runs its course, whether favourably or fatally; and, as in all climates mercury interferes not with other remedies, Dr. M. thinks its administration ought never to be omitted.

"I undoubtedly have had many examples of the good effects from mercury in the cure of this disease. Four grains of calomel given two or three times a-day, with three or four drachms of the ointment well rubbed on the neck and spine night and morning, I believe to be excellent practice. A much larger quantity of the ointment may be used on different parts of the body; indeed, the more continued the friction, the better. The constitution labouring under this disease, will mostly appear as proof against the usual effects of this medicine; but when salivation can be brought about, it will in a great majority of cases be found to be attended with the happiest consequences. Allowing the spontaneous salivation which sometimes occurs, to be more the effect than the cause of the cure, still we would be inclined to throw in large quantities of mercury, merely with a view of bringing on any different action in the system."

The sublimate of mercury with scammony or jalap as a purge is also recommended by our author.

*Opium.* This appears the sheet anchor of our author in this disease. He has met with more than a dozen cases where the cure of tetanus could be fairly attributed to this medicine; and he has met with no instance of recovery in which he did not conceive that it bore a principal part. It must be given, however, in very large doses, the system under tetanus being little affected by doses of opium that in other circumstances would produce striking effects.

"A practitioner," says Dr. M. "for whose acuteness and discernment I have great respect, gave to an old man, in my presence, who was in an incipient stage of this disease, about *half an ounce* of tincture of opium in four ounces of rum, as *a first dose*, directing, at the same time, the spirit to be frequently repeated, and the man got perfectly over the complaint in a few days." 57.

Dr. M. directs that an adult should commence with one hundred drops of the tincture (bowels being opened), increasing each succeeding dose one-third every two hours, unless sleep or stertor in the breathing ensue; ordering at the same time, wine or ardent spirits, in as large quantites as the patient can be induced to swallow. A pint of spirits, or double that quantity of wine in the twenty-four hours will not be too much. Tincture of opium is also to be rubbed on the spine

*The Warm Bath* is regarded by our author in a favourable point of view. It has afforded much present relief on several occasions under his own eye, where the spasmodic twitchings were frequent and troublesome. He depends very little on it, however, and justly observes, that the exertion or movement which the patients must undergo, in order to get into the bath, will often more than counterbalance any good effects that can be expected from it. Patients are so alive to all external impressions, that the least exertion is often sufficient to excite violent spasms. On this account, the patient should be kept as quiet as possible, and very few questions asked him. The chamber should be kept darkened, and every thing tending to excite mental exertion avoided.

*Blisters,* though recommended in high terms by a few medical practitioners, can only be looked upon in the light of adjuvants. The course of the spine appears the best site of their application.

*Bark and Wine.* Dr. M. recommends, that during the exhibition of opium, large quantities of wine or diluted alkohol be administered, in order to second its effects.

*Cathartics* are not considered by our author as of any material use; and the same may be said of venæsection.

We shall give the author's plan of treatment in his own words.

"The bowels should be kept as free as possible. We must endeavour to bring about an operation every twelve hours. This, even by the aid of strong cathartics, or purgative injections, will be found very difficult to be obtained; the sphincter ani sometimes scarcely admitting the introduction of a glyster-pipe, and the exhibition of the strongest purgatives may often be attended with little or no effect. Sulphate of soda, jalap and calomel, scammony, pil. aloes cum colocythide, &c. are as proper for this purpose as any other, aided by stimulating clysters, such as a solution of muriate or sulphate of soda, with olive oil; the resin of turpentine, suspended by the yolk of an egg; solutions of soap, &c. I have found it, on two or three occasions, impossible to open the bowels freely, till after large quantities of opium had been taken, which seemed to bring about a general relaxation; or until the system had been evidently under the influence of mercury; and, indeed, these are the two medicines on which we are to place the greatest confidence, in the treatment of this disease: they must be given, however, as before remarked, in large doses, and frequently repeated. I once gave a patient, who is, I believe, still living, ten grains of opium and twenty of calomel, in pills, and five ounces of tincture of opium, in wine, all in the space of twelve hours.

"Next to opium, I certainly look on the preparations of quicksilver as the most valuable. Large quantities of the ointment may be rubbed in on the spine, neck, legs, &c. with repeated doses of submuriate internally. Wine and ardent spirits should be given freely; indeed, the constitution here appears as insensible to their usual effects, as to those of opium; and quantities, which in a state of health, would produce stupid intoxication, now neither exhilirate the spirits, nor disturb that serenity of mind so conspicuous throughout the disease.

"The *warm bath* will often be found a useful auxiliary; when we expect to derive advantages from it, the vessel used should be so capacious, as to allow the patient to be as little confined as possible, and the water should be sufficient to cover the shoulders completely. I have found a common rum puncheon sawed across at the centre, very convenient for this purpose.

"I have generally used blistering plasters, but confess I have never experienced much benefit from their application.

"When the disease is conquered, the patient should take wine and bark for many weeks." p. 70.

We shall abridge a few cases in elucidation of our author's mode of treatment in this disease. The first and second were fatal—the cold affusion was used. These we shall not notice. The third is more interesting.

A Negro, ætat. 35, slender habit, came into hospital, 13th of March, 1810, complaining of pain in his neck; restlessness and feverishness at night; his pulse was 76; abdomen tense, skin warmer than usual, countenance natural. He took two ounces of salts, with a grain of emetic tartar, which operated three times.

14th. Little or no sleep; neck more painful; he can scarcely open his mouth, his jaws being stiff and uneasy; his abdomen is hard, umbilicus drawn in. Took eight grains of calomel, and three drachms of laudanum in two ounces of spirits every two hours. Rubbed in the mercurial ointment on the spine, and used the warm bath every four hours.

15th. Omnia exacerbata; muscles of the back, thighs and legs rigid; bowels confined; pulse 80; skin moist. Continued the mercurial ointment and the warm bath; took half an ounce of laudanum every hour, with a purgative enema three or four times a-day.

16th. The injections produced the desired effect; swallows with difficulty; has had spasms every ten minutes during the night, so violent that the occiput and heels only touch the bed. Five drachms of laudanum every hour; four grains of calomel three times a-day. Continues the mercurial frictions and the warm bath.

17th. Deprived of sleep by the spasms which are more frequent, and so violent as almost to throw him out of bed. Skin covered with perspiration; pulse, in the absence of spasm, 84.

18th. Has taken the opium in spirits regularly, with about a bottle of Madeira wine. The spasms are less frequent, and he has a little sleep at intervals. A copious alvine evacuation at seven in the morning. Continuentur omnia.

19th. Spasms less frequent and violent, abdomen softer, and jaws opened farther than yesterday. An increased flow of saliva, but no mercurial fœtor; pulse 88 and not so full; he seems in every way better.

20th. The calomel to be omitted, the laudanum, wine and warm baths to be continued.

21st. Can swallow with tolerable ease; slept two hours in the night; spasms nearly gone; abdomen feels natural. From this till the 25th, the laudanum was gradually left off, when he began to take bark and wine, and in a month was perfectly recovered.

Case 5. July 10. A Negress, ætat. 28, had been ailing two days with a tightness in her jaws, and difficulty in swallowing. She had taken a dose of salts, and rubbed her neck with camphorated spirits to no effect. Countenance is strongly characteristic of the disease; pain shooting from the epigastric region towards the spine. Ordered her eight grains of calomel, with twelve of scammony, and an enema. After three hours, to take two drachms of laudanum in two ounces of spirits, and to repeat the dose every two hours. About twelve she had a copious stool; skin dry; pulse 86; muscles of the neck rigid. At 6, P. M. ordered her three drachms of laudanum in rum every second hour; the spine to be rubbed with ung. hydr. and the epigastric region with laudanum; to take a glass of Madeira frequently.

11th. Had taken the laudanum as ordered; got two clysters, the last of which operated once; the head and shoulders are rigidly drawn back on the least attempt at motion; pain of the epigastric region severe; spasms of the muscles of the upper part of the body occur every six or eight minutes. Half an ounce of tinct. opii every two hours; frictions continued; Madeira wine ad libitum.

12th. Slept a little last night; the laudanum regularly continued; skin softer than yesterday; abdomen not so tense; legs and thighs occasionally stiffly extended; great pain in the epigastrium. Administered six drachms of laudanum immediately; and then the former dose to be continued as before. The six drachm dose was given in three ounces of brandy. She now also took two grains of opium, and two of calomel every hour. Frictions continued. Wine ad libitum.

13th. Had a small operation this morning; slept an hour at intervals, during the night; swallowing is performed much easier than before; there is a slight discharge of saliva; passed about a pint of pale urine yesterday evening; skin warm and moist; pulse 85. To take an ounce of tinct. opii in three ounces of brandy every two hours. Pills continued.

14th. Is much easier; got two purgative injections last night, which operated this morning; the legs are now capable of voluntary motion, and swallowing is easier accomplished; the lips are relaxed; salivation is present. Contin. omnia medic.

15th. Has taken in the twenty-four hours, *eight ounces* of laudanum, with twenty grains of opium and as much calomel; abdomen softer; salivation continues moderate; can swallow with comparative ease, and makes every effort to swallow her medicine. Omit the pills; continue tinct. opii et frictions; wine ad libitum.

16th. Appears much better; had three stools yesterday; took some soup; the jaws can be opened more freely; skin covered with perspiration. Cont. medic. ut heri.

17th. All the muscles much relaxed; spasms disappeared; pulse 90 and full; skin warm and moist. To take two pints of wine to-day; half an ounce of laudanum every two hours in brandy.

18th. Slept two hours last night; jaws and neck are little affected; complains of pain under the ensiform cartilage, on pressure; salivation diminished; has taken four ounces of laudanum since yesterday and a bottle of wine; had a stool during the night. Continue the wine and two drachms of laudanum every two hours.

19th. All the symptoms favourable, but attended with great debility; to take a wine glass of the following mixture every hour, with two drachms of laudanum night and morning. ℞. Cinchon. pulv. ℥ij; vin lusitan Oij M.

21st. Debility continues; has taken food. She gradually left off the laudanum, but continued the bark and wine for two weeks, when she seemed quite recovered.

*Remarks.* "In this case, the opium was given in larger quantities than in any I ever met with. The patient (to my knowledge) enjoyed excellent health for two years afterwards."

Case 6. *(Symptomatic.)* February 3. A Negro having received a slight flagellation a few days since; came yesterday morning into the hospital, with severe pain in the small of his back and loins; his head and shoulders are frequently drawn back, as in pain, which he refers to the small of his back; countenance characteristic; pulse healthy; had a small motion this morning in consequence of a laxative taken last night; slept ill for two or three nights last; the back part of his neck stiff and painful; can open his jaws as usual. Ordered ten grs. of calomel with ʒß cathartic extract in six pills; three to be taken immediately, and repeated in four hours.

4th. Pills have operated briskly; cannot open his mouth so freely; spasms occur every ten or twelve minutes, by which his head and shoulders are much drawn back; during the presence of the spasms, the thighs and legs are suddenly extended; respiration laborious; heat of the body rather more than natural. To take ʒiij of laudanum every two hours, and grs. iv of calomel three times a-day, with the warm bath.

5th. Has taken the medicines regularly; slept very little last night; has been twice in the bath, and experienced relief; jaws during the spasms (which occur every five or six minutes) closely contracted, combined with opisthotonos; abdomen tense, and hard. I now gave him half an ounce of laudanum in four ounces of brandy, and directed the former medicines to be continued; the warm bath to be repeated three times a-day, mercurial frictions to the neck and spine; and a purgative enema every two or three hours.

6th. Has used the bath twice to day, and seems relieved while in it; has taken the medicine regularly; had a copious evacuation in the night; skin perspirable; countenance extremely characteristic; thinks he can swallow easier. Contin. omn. medic.

7th. Slept a little last night; had a motion after the bath this morning; says he does not "feel his body so tight;" skin covered with perspiration; salivation has commenced. Perstat ut heri.

8th. Appears much better; can swallow with tolerable ease; the head and shoulders still further back than natural; took food this morning; slight spasms occur every quarter of an hour. Omit the calomel. Continue the laudanum.

9th. Is every way better; head and shoulders continue contracted; slept three hours last night; has taken half a bottle of Port wine; abdomen softer; had a natural stool this morning. Omit the bath; ʒij of laudanum every two hours; wine ad libitum.

10th. The disease is subdued; salivation continues; slept half the night; took some soup to day. The tincture of opium was gradually left off; he continued the bark and wine till the 20th, when he left the hospital quite well.

Case 7. April 10th, 1812. A Negro came into the hospital this day, complaining of dysphagia; pain under the sternum; tightness about the jaws; countenance peculiar. About ten days ago he trod on an old nail, which penetrated the heel about a quarter of an inch; the wound bled a few drops; was poulticed, but attended with little or no suppuration, and has given but little pain or uneasiness since. Made an opening into it with a lancet, and there issued from it about two tea spoonfuls of a dark purulent matter. Applied a hot poultice, which was renewed every four or five hours. Two scruples of jalap and eight grains of calomel to be taken immediately; an enema injected. 3 P. M. Has had two plentiful evacuations, and passed urine. Spasmodic twitchings have commenced; much pain under the sternum; pulse 86. Heat of skin above the natural standard. ℞. Opii ʒß submur. hyd. ℈ij fiant pilulæ decem. Capiat unam tertiis horis. Blisters to the sternum. An enema injected twice a-day.

11th. Little sleep last night; thinks the blister has relieved the pain; had a stool last evening. Has taken five pills since yesterday, and a bottle of wine. Spasmodic twitchings more frequent; tightness of the jaws not increased. Pills to be continued, and to take two drachms of tincture of opium in two ounces of diluted alcohol every two hours.

12th. Got an hour's sleep last night; spasms very frequent; can swallow rather easier; skin covered with perspiration. To continue the pills and tincture, &c. as before. The spine to be rubbed with mercurial ointment.

13th. Has taken the medicines regularly; jaws evidently relaxed; pain at the ensiform cartilage much relieved; the salivary glands affected with mercury; complains of his legs being very itchy.

14th. Seems better in every respect; little or no return of spasms; abdomen natural. Omit the pills; to take ℥ß of laudanum every three hours in brandy.

15th. Strong mercurial fœtor on the breath; the legs continue itchy, and are covered with a rough, sandy eruption; pulse 90 and small; has taken a little soup to day. Three drachms of tinct. opii three times a-day.

17th. The disease has left him; he is much emaciated, and reduced in strength; the eruption on the legs is nearly gone, though they still continue to itch. He remained till the effects of the mercury were gone off, when he commenced the use of tonics, and ultimately retired perfectly recovered.

Although from the foregoing Analysis our readers will be pretty well able to form an estimate of the merits of Dr. Morrison's work, yet we shall add a few observations, partly the result of our own experience; partly the remarks of a friend, in whose judgement we have the fullest confidence.

Dr. M. appears to have erred in considering idiopathic tetanus almost as fatal as traumatic. He also seems to look on tetanus generally, as a less serious malady than the experience of others has found it. We are inclined to think, that Dr. Morrison's experience is chiefly drawn from the sub-acute, or chronic forms of the disease, which are by no means so dangerous as the acute. Our experience does not coincide with that of Dr. M. respecting the alvine secretions, which he represents as *natural* in general. He also seems to place too little reliance on purgatives.

We may here be allowed to make the *amende honorable* to Dr. Dickson, of Clifton, for some errors and oversights in our review of his paper in the Medico-Chirurgical Transactions, upon the great utility of purgatives in preventing tetanic affections. Upon more mature reflection, we are convinced that that gentleman is right in placing *torpor of the bowels* as a very general antecedent, if not an occasional cause of tetanus; and hence, that his prophylactic suggestions are of the utmost importance. "*Un j'ai tort vaut mieux q'une mille repliques ingenieuses.*"

That distinguished physician justly conceives, that a phænomenon so invariably preceding the attack of idiopathic tetanus, cannot but be regarded with a suspicious eye, and ought to be a beacon to warn us of approaching danger. Upon the whole, this little volume is highly entitled to the general perusal of the profession.

